# Screening and identification of genes associated with flight muscle histolysis of the house cricket *Acheta domesticus*

**DOI:** 10.3389/fphys.2022.1079328

**Published:** 2023-01-11

**Authors:** Ying Lu, Zizhuo Wang, Fei Lin, Yuqing Ma, Jiangyan Kang, Yuying Fu, Minjia Huang, Zhuo Zhao, Junjie Zhang, Qi Chen, Bingzhong Ren

**Affiliations:** ^1^ Key Laboratory of Economical and Applied Entomology of the Education Department of Liaoning Province, College of Plant Protection, Shenyang Agricultural University, Shenyang, China; ^2^ Jilin Provincial Key Laboratory of Animal Resource Conservation and Utilization, Key Laboratory of Vegetation Ecology, Ministry of Education, School of Life Sciences, Northeast Normal University, Changchun, China; ^3^ College of Life Sciences, Jilin Normal University, Siping, China; ^4^ Engineering Research Center of Natural Enemies, Institute of Biological Control, Jilin Agricultural University, Changchun, China

**Keywords:** flight muscle histolysis, cricket, transcriptomics, FABP, RNA interference

## Abstract

**Introduction:** Flight muscle histolysis, as an important survival strategy, is a widespread phenomenon in insects and facilitates adaptation to the external environment in various insect taxa. However, the regulatory mechanism underlying this phenomenon in Orthoptera remains unknown.

**Methods:** In this study, the flight muscle histolysis in the house cricket *Acheta domesticus* was investigated by transcriptomics and RNA interference.

**Results:** The results showed that flight muscle histolysis in *A. domesticus* was standard and peaked within 9 days after eclosion of adult crickets, and there was no significant difference in the peak time or morphology of flight muscle histolysis between males and females. In addition, the differentially expressed genes between before and after flight muscle histolysis were studied, of which AdomFABP, AdomTroponin T and AdomActin were identified as candidate genes, and after injecting the dsRNA of these three candidates, only the downregulated expression of AdomFABP led to flight muscle histolysis in *A. domesticus*. Furthermore, the expression level of *AdomFABP* was compared between before and after flight muscle histolysis based on RT-qPCR.

**Disscussion:** We speculated that *AdomFABP* might play a role in the degradation of flight muscle by inhibiting muscle development. Our findings laid a molecular foundation for understanding the flight muscle histolysis.

## 1 Introduction

In insects, flight muscles are responsible for that power flight movement (Snelling et al., 2012; Ding et al., 2014; Lu et al., 2020). Numerous studies on flight muscle have been reported in various insect taxa, such as oriental armyworm (*Mythimna separata*) and some species of locusts (*Locusta migratoria manilensis*, *Oedaleus asiaticus* and *Calliptamus italicus*) (Luo, 1996; Liu et al., 2008; Dou et al., 2017; Han et al., 2020). The research mainly focused on three aspects. The relationships between flight muscle and flight ability and between energy accumulation and consumption during flying (Liu et al., 2008; Dou et al., 2017; Cao and Jin, 2020). The relationship between flight muscle development and reproductive system development (Roff and Fairbairn, 2007; Zhao et al., 2010; Chang et al., 2021; Guo et al., 2022), especially the trade-off between flight capability and reproduction in female insects (Ge et al., 2021). (3) The effects of insecticides on flight muscles in the context of pest control (Liu et al., 2017).

Flight muscle histolysis is an important survival strategy for many insects; it allows them to conserve and utilize compounds and energy and adapt to changes in the external environment after their flight muscles have fully developed (Kobayashi and Ishikawa, 1994). Flight muscle histolysis occurs not only after the end of migration but also when wing dimorphism arises, as observed in the cricket *Velarifictorus asperses* and the butterfly *Pieris napi* (Stjernholm and Karlsson, 2008; Zeng et al., 2012; Zhang et al., 2019). Therefore, studying flight muscle histolysis can provide a better understanding of migration patterns, population dynamics, reproductive signals and wing dimorphism in insects.

To date, many studies have focused on the physiological basis of insect flight muscle histolysis through investigations of muscle composition, morphological differences between before and after flight muscle histolysis, physiological significance and other aspects (Stjernholm and Karlsson, 2008; Gibbs and Van Dyck, 2010; Chaulk et al., 2014; Liu et al., 2017; Matte and Bliien, 2021; Kurihara et al., 2022). For example, a decrease in protein content in flight muscle of the pea aphid *Acyrthosiphon pisum* has been found to occur simultaneously with flight muscle histolysis (Kobayashi and Ishikawa, 1994). Other studies have focused on the physiological and molecular mechanisms underlying flight muscle histolysis by investigating the factors affecting flight muscle histolysis, the relationship between flight muscle histolysis and apoptosis, and other aspects (Tanaka, 1994; Domingo et al., 1998; Fyrberg et al., 1998; Nongthomba et al., 2001; Wu and Haunerland, 2001; Zera and Cisper, 2001; Wu et al., 2002; Gajewski et al., 2006; Azizi et al., 2009; Feng et al., 2019; Katti et al., 2021; Bai et al., 2022). For example, the flight muscle histolysis in both the fire ant *Solenopsis invicta* and the wheat aphid *Sitobion avenae* occurs through apoptosis (Azizi et al., 2009; Feng et al., 2019). Juvenile hormone (JH) can affect the development and degradation of flight muscle in *Modicogryllus confirmatus* (Tanaka, 1994), *Gryllus firmus* (Zera and Cisper, 2001), and *Acyrthosiphon pisum* (Bai et al., 2022). Mitochondria, as organelles supplying energy to the body, can also influence and regulate the formation and degradation of flight muscles by regulating their own fusion and fission (Katti et al., 2021). In addition, there are studies that say due to troponin and actin are important structural proteins involved in the assembly and regulation of myofibrillar fibers, so as to the expression of genes encoding troponin and actin can determine the structural and functional integrity of flight muscles (Domingo et al., 1998; Fyrberg et al., 1998; Nongthomba et al., 2001; Gajewski et al., 2006). And lipid-related genes that regulate the expression of lipid substances also play a very important role in the degradation of flight muscle, because of lipids are among the necessary energy substances for insect flight activities (Wu and Haunerland, 2001; Wu et al., 2002). However, since the degradation of flight muscle is affected to varying degrees by a variety of factors, the regulatory pathways of flight muscle histolysis are poorly understood.

The house cricket *Acheta domesticus* Linnaeus (Orthoptera: Gryllidae) is widely distributed worldwide and has a wide range of food habits. *A. domesticus* is an excellent and often used model organism due to its many advantages, such as its small size, ease of rearing, short life cycle, nutrient richness, lack of diapause, high tolerance and ease of handling (Clifford et al., 1977; Crocker and Hunter, 2018). These advantages make this species ideal for research on the development and utilization of edible and forage insects (Patton, 1978; Grossmann et al., 2021; Khatun et al., 2021), immune regulation (Piñera et al., 2013), adult neurogenesis (Cayre et al., 2007), and other topics (Nelson and Nolen, 1997; Fuciarelli and Rollo, 2020; Deban and Anderson, 2021; Li and Rollo, 2021). Moreover, after *A. domesticus* eclosion, their flight muscles degraded, and some but not all individuals shed their hind wings, resulting in wing dimorphism. Therefore, *A. domesticus* is an ideal model organism for studying insect wing dimorphism as well as flight muscle histolysis (Chen et al., 2019). Studies on *A. domesticus* have revealed that flight muscle cell apoptosis could be regulated by hormones and lead to histolysis (Oliver et al., 2007); however, the regulatory pathways and signaling pathways that are activated during flight muscle histolysis and the functions of key genes during this process in *A. domesticus* remain unknown. Therefore, the aim of our study was to investigate the molecular mechanism underlying flight muscle histolysis in the house cricket *A. domesticus* through transcriptomics and RNA interference (RNAi). Our findings provided new insight into flight muscle histolysis in Orthoptera at the gene level.

## 2 Materials and methods

### 2.1 Insects

The house cricket *A. domesticus* was obtained from a commercial colony (Beijing, Sanyuanxishuai, China) and reared in the entomology laboratory of Northeast Normal University starting in early 2015 (Changchun, China). After diapause at 2°C–4°C for approximately 1 week, the eggs were incubated in an artificial climate chamber (BIC 300, Boxun, Shanghai) at 25°C. After hatching, the nymphs were reared in an insect culture chamber at 30°C and 30% relative humidity in the dark. Before eclosion, the nymphs were reared in large rearing boxes (53 cm × 38 cm × 33 cm) at a density of 600∼700 crickets per box. The nymphs were fed daily with 600 g of carrots and 30 g of mixed nutrient powder (composed of 35% crude wheat bran, 20% corn starch, 20% soybean flour, 15% skim milk powder, 5% active dry yeast and 5% animal liver meal) per box. After eclosion into adults, the crickets were maintained in small rearing boxes (17 cm × 11.5 cm × 6 cm), each containing 20 individuals at a female:male ratio of 1:1. Each box of crickets was provided with 100 g (1 cm^3^) of carrots mixed with 5 g of nutrient powder each day.

### 2.2 Study on flight muscle histolysis of *A. domesticus*

The newly eclosed adults were housed in rearing boxes (17 cm × 11.5 cm × 6 cm), each containing 20 individuals at a female:male ratio of 1:1. In this study, 30 boxes containing a total of 600 adult crickets (ten boxes in a set, with three replicates) were provided with the same adult diet as described above. To maintain a constant rearing density, spare individuals housed under similar conditions were used to replace the dead crickets in the experience groups.

Flight muscle histolysis in *A. domesticus* was observed daily on 0 to 11th days after eclosion, and 10 individual crickets with similar body sizes were used for observation each day. The crickets selected for observation were frozen at −20°C for 10 min and then thawed at room temperature (25°C) for 2 min. After that, the fore and hind wings were all removed with curved forceps under a stereomicroscope. Then, forceps were used along the end of the abdomen, and all the integument was completely removed up to the pronotum, exposing the dorsal longitudinal muscles (DLMs). The changes in the dorsal and abdominal longitudinal muscles from before to after flight muscle histolysis were evaluated. Each side of the DLMs was approximated as a rectangle. The length, width (at the widest point) and height of each of the left and right DLMs were measured by vernier caliper in this study. Then, the mean volume of the left and right DLMs was calculated and used as the volume of the flight muscle of the house crickets. Finally, according to the daily observations of the DLM volumes of all of the house crickets, the volume changes in the degradation process of flight muscle were obtained. To visualize the volume variation from before to after flight muscle histolysis, this study divided the degradation process of flight muscle of *A. domesticus* into four stages: 0–2 days for non-degradation (Stage I), 3–5 days for early degradation (Stage II), 6–8 days for late degradation (Stage III), and 9–11 days for total degradation (Stage IV). Thirty individual crickets with similar body sizes were used for volume determinations each day, observations were conducted on 3 days for each stage, and a total of 90 individuals were used for volume determinations in each stage.

### 2.3 Transcriptome sequencing of *A. domesticus* flight muscle

#### 2.3.1 Collection of flight muscle samples

Based on the degradation of flight muscle observed in the previous experiment, two sampling stages were determined: before flight muscle histolysis (day 0 after eclosion, abbreviated as O1) and after flight muscle histolysis (day 10 after eclosion, abbreviated as R1). DLM samples were collected at these two time points using the sampling method described in Section 2.2. After removal of the trachea, the DLMs were cleaned in PBS buffer and then placed in 2 mL sealed lyophilized tubes for storage. After labeling, the lyophilized tubes were frozen in liquid nitrogen for 30 min and finally stored in a −80°C freezer until RNA extraction. A total of at least 2000 female house crickets were raised for sampling in two periods (O1 and R1). All of the house crickets used for transcriptome sequencing were female.

#### 2.3.2 RNA extraction and sequencing of flight muscle samples

According to the above two sampling stages (O1 and R1) and the sampling requirements to determine the developmental status of the DLMs, RNA extraction of the DLM samples was carried out in batches. Total RNA was extracted from each cricket by TRIzol reagent (Invitrogen, Carlsbad, CA, United States). The quality and concentration of the total RNA in each sample were assessed by preliminary purification. Only the samples of extracted RNA that met the sequencing conditions (based on the electrophoresis results) were mailed to Beckman Coulter, Inc. (Biomarker, Beijing) for transcriptome sequencing. After the mRNA was highly purified with magnetic oligo(dT) beads, the NEBNext Ultra RNA Library Prep Kit with six-base random primers (random hexamers) was used for RNA-seq library preparation. Library purification was performed by using AMPure XP Beads. A HiSeq 4000 platform (Illumina, New England Biolabs, Ipswich, MA, United States) was used for high-throughput sequencing with the PE150 read type.

### 2.4 Data analysis

#### 2.4.1 Sequence assembly and functional annotation

High-quality clean reads were obtained by removing duplicated sequences, adaptor sequences, low-quality reads and ambiguous reads (reads with unknown ('N') nucleotides). Transcriptomes were *de novo* assembled separately using Trinity (http://trinityrnaseq.sourceforge.net/). Assembly version: r20131110. Assembly parameters: the shortest length of the assembled contig is 200; the length of the inserted contig is 500; other values are the default. A total of 36,163 Unigene were obtained by assembly, and the N50 of Unigene was 2,274, showing high assembly integrity. The unigenes were first annotated in the National Center for Biotechnology Information (NCBI) non-redundant protein sequence database (NR), the protein sequence database Swiss-Prot; and the Gene Ontology (GO), Clusters of Orthologous Genes (COG), Eukaryotic Orthologous Groups (KOG), evolutionary genealogy of genes: Non-supervised Orthologous Groups (eggNOG), and Kyoto Encyclopedia of Genes and Genomes (KEGG) databases. In this study, 13,449 annotated unigenes were obtained by selecting BLAST parameter E-value not greater than 1e-5 and HMMER parameter E-value not greater than 1e-10. Then, BLAST searches of the NR database were performed to explore the candidate genes related to flight muscle histolysis in *A. domesticus*.

#### 2.4.2 Differential gene expression analysis

Gene expression was determined by the fragments per kb per million reads (FPKM) method. Differential expression analysis was performed with the help of DESeq (http://www.bioconductor.org/packages/release/bioc/html/DESeq.html), followed by a false discovery rate (FDR) test using the Benjamini-Hochberg (BH) procedure and *p*-value correction for the obtained datasets. The *p*-value correction was applied to Pearson’s correlation analyses to confirm the reliability of the replicates. In addition, the analysis of differentially expressed genes (DEGs) between before and after degradation of *A. domesticus* flight muscle was performed by comparing data between the two stages (O1 and R1).

### 2.5 Identification and validation of candidate genes associated with flight muscle histolysis in *A. domesticus*

#### 2.5.1 Identification of the candidate genes

The transcriptome was sequenced before and after flight muscle histolysis based on the flight muscle histolysis pattern in *A. domesticus*. DEGs were screened according to the transcriptome sequencing results, focusing on the top 200 upregulated genes and the top 200 downregulated genes. Then, functional exploration was first carried out for genes that have been shown to be associated with flight muscle in previous studies among these DEGs and RNAi was applied to verify their functions.

#### 2.5.2 Quantitative reverse transcription polymerase chain reaction (RT-qPCR) of the candidate genes

Twelve samples from different life periods, i.e., the last nymph stage and 0–10th days after eclosion, were used to explore the expression trends of the candidate genes. Five females were selected at each time point for flight muscle dissection and sampling, each as a group of biological replicates. The sampling method was the same as that described in Section 2.3.1. Then 5 groups of replicate samples of each period were mixed to extract RNA. RNA was extracted from flight muscle specimens using TRIzol reagent (Invitrogen, Carlsbad, CA, United States) in batches, and the quality and concentration of RNA were determined. After RNA quality was confirmed, 2 μg RNA was quantified and reverse transcribed into cDNA. The expression of candidate genes was measured by RT-qPCR, which was conducted with a StepOnePlus real-time polymerase chain reaction (PCR) assay system (Bio-Rad, Hercules, CA, United States) and TransStar Tip Top Green qPCR Supermix (TransGen Biotech, Beijing, China). 18S rRNA was used as the reference gene. The PCR conditions were as follows: 94°C for 30 s, followed by 40 cycles of 94°C for 5 s, 60°C for 15 s, and 72°C for 10 s. Then, conditions of 95°C for 15 s, 60°C for 30 s, and 95°C for 15 s were used to construct dissociation curves. The RT-qPCR data were analyzed using the 2^−ΔΔCT^ method based on at least three technical replicates. All primers used in this study were designed using Primer Premier 5. The efficiencies of the primers were measured before RT-qPCR. The primers for the candidate genes used in the RT-qPCR assays are listed in Supplementary Table S1.

#### 2.5.3 RNAi of candidate genes

To further identify the genes that determine flight muscle histolysis of *A. domesticus*, DEGs obtained from comparison of the O1 and R1 transcriptome data (before and after flight muscle histolysis) were comprehensively analyzed. The DEGs, namely, *AdomFABP*, *AdomTroponin T* and *AdomActin*, were functionally validated by using double-stranded RNA (dsRNA) targeting green fluorescent protein (GFP) dsGFP as a negative control. The dsRNA was injected into crickets on day 0 after eclosion. The primers for the candidate genes used for RNAi are listed in Supplementary Table S2.

The specific procedure was as follows. *AdomFABP*, *AdomTroponin T* and *AdomActin* and dsGFP were synthesized from the corresponding PCR fragments and amplified with upstream and downstream primers containing the T7 promoter. Due to the high FPKM value of candidate genes at the O1 stage, RNA reverse transcription at this stage was used to produce a cDNA template. dsRNAs were synthesized using the MEGAscript RNAi kit (Ambion, Austin, TX, United States). The synthesized dsRNAs were purified by precipitation with lithium chloride. Each dsRNA pellet was dissolved in 50 μL of nuclease-free water, quantified by a NanoDrop 2000 spectrophotometer and 1% agarose gels with GoldView Nucleic Acid Stain (Dingguo, China) and stored at −80°C before use. dsRNA was injected into crickets on day 0 after eclosion. A final amount of 6 μg targeted dsRNA in a 4 μL volume was injected at the second ventral segment of the abdomen by using a Nanoliter 2000 injector (World Precision Instruments, Sarasota, FL, United States). The dsRNA for each gene was injected into 120 crickets, which were subsequently returned to their original boxes and maintained under the same conditions described above to observe flight muscle histolysis. Six days after each injection, RNA was extracted from the samples, and 2 µg of RNA was quantified and reverse transcribed into cDNA; the interference effect was evaluated using RT-qPCR. RT-qPCR was performed by using the StepOnePlus Real-Time PCR Detection System (Bio-Rad, Hercules, CA, United States) and TransStar Tip Top Green qPCR Supermix (TransGen Biotech, Beijing, China). 18S rRNA was employed as an internal control. The PCR conditions were as follows: 94°C for 30 s, followed by 40 cycles of 94°C for 5 s, 60°C for 15 s, and 72°C for 10 s, followed by melting curve analysis. The RT-qPCR data were analyzed by the 2^−ΔΔCT^ method, which is based on at least three repeats. All of the primers employed in this study were designed with Array Designer 4.3 (PREMIER Biosoft, Palo Alto, CA, United States). The efficiencies of the primers were tested before RT-qPCR. The primers for candidate genes used for testing the RNAi effect are listed in Supplementary Table S3.

## 3 Results

### 3.1 Flight muscle histolysis in *A. domesticus*

**FIGURE 1:**
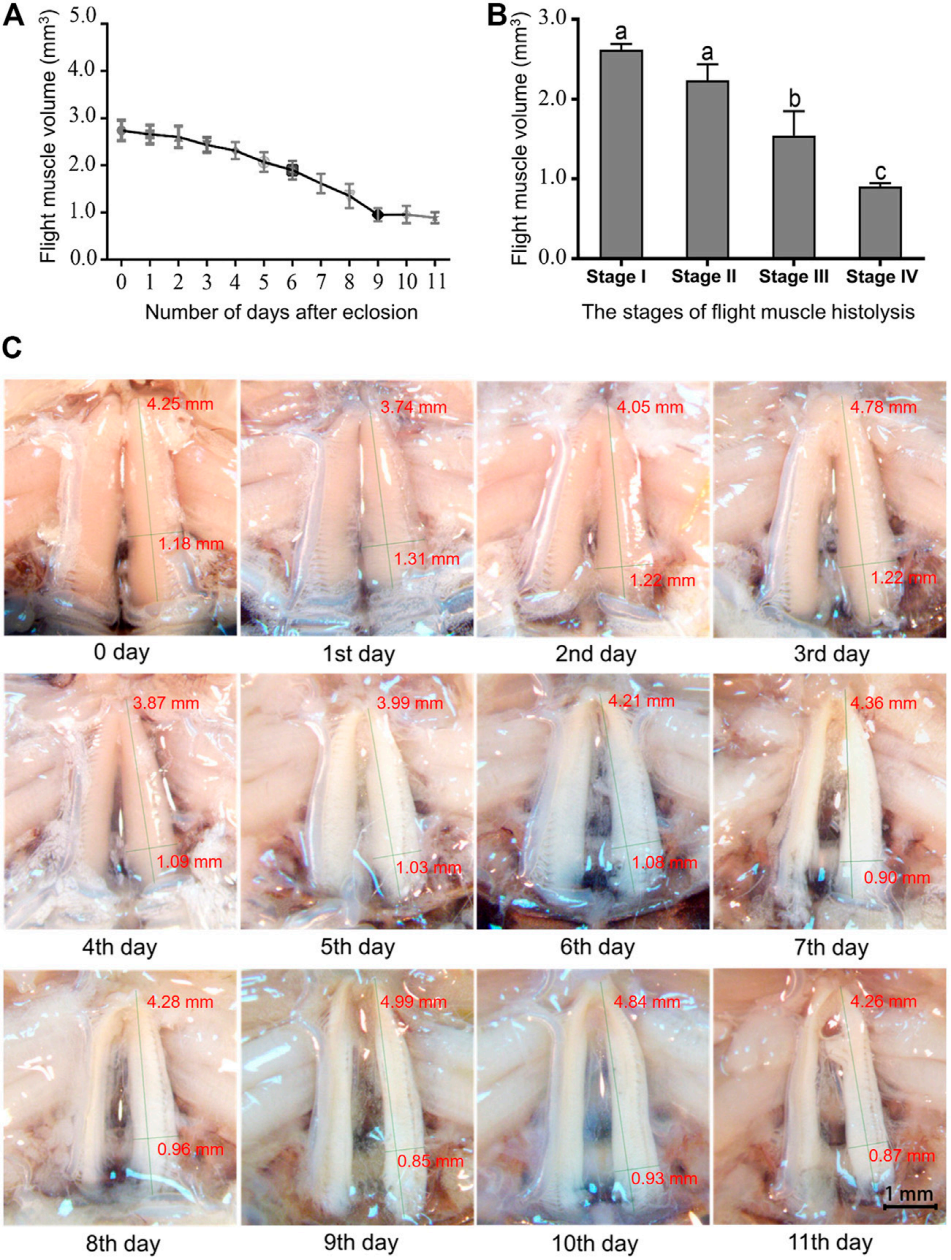
Changes in the dorsal longitudinal muscle during flight muscle histolysis in *A. domesticus*. **(A)** Variation in dorsal longitudinal muscle volume during flight muscle histolysis; **(B)** Analysis of the significant of dorsal longitudinal muscle volume changes at various stages of flight muscle histolysis. Stage I: 0–2 days of no degradation of the flight muscle; Stage II: 3–5 days of early degradation of the flight muscle; Stage III: 6–8 days of late degradation of the flight muscle; Stage IV: 9–11 days of total degradation of the flight muscle. **(C)** Morphological changes in the flight muscle during days 0–11 after emergence.

**FIGURE 2:**
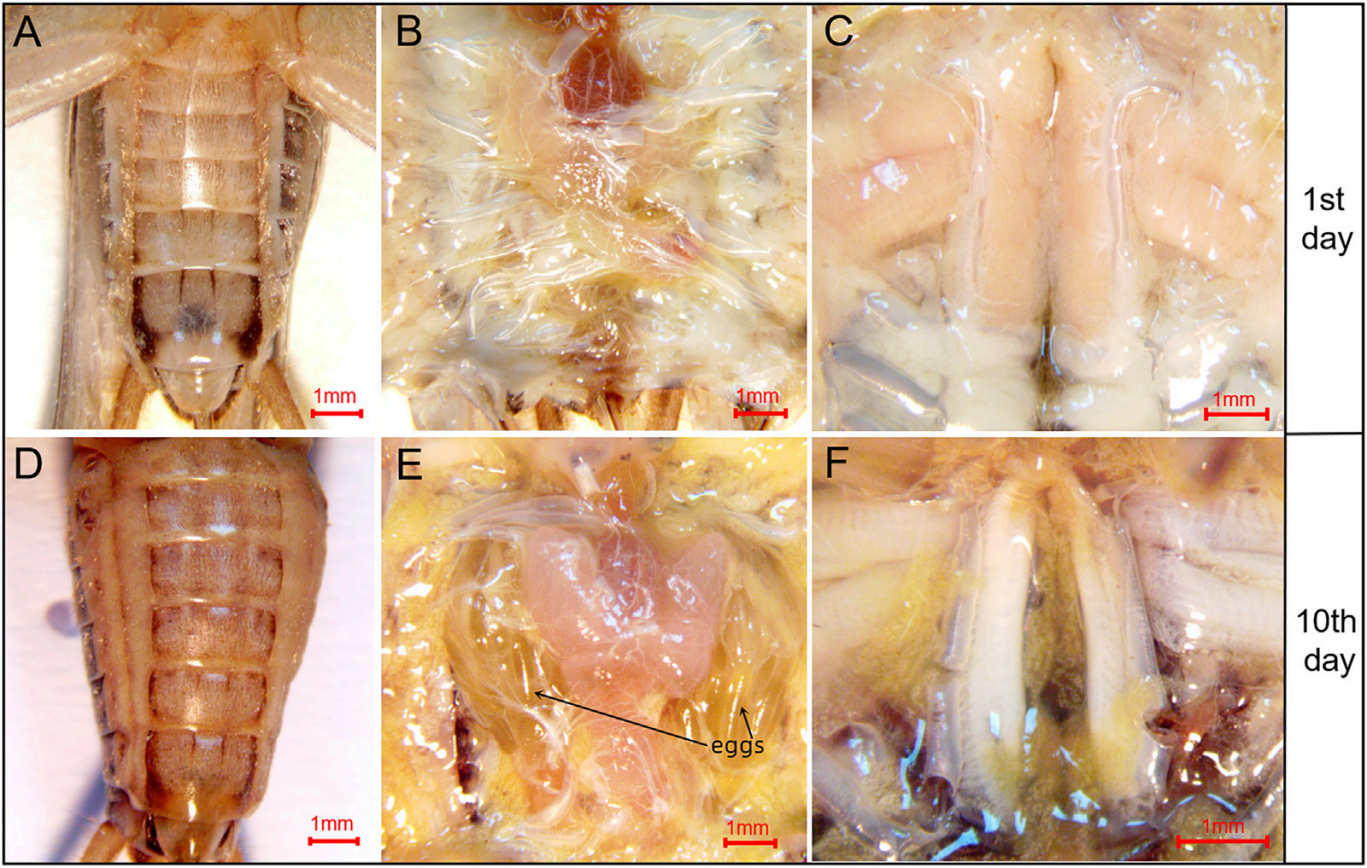
Morphological changes in the abdomen of *A. domesticus* females from before to after flight muscle histolysis. **(A)** The anatomy of female crickets on the 1st day after eclosion; **(B)** The abdominal anatomy of female crickets on the 1st day after eclosion; **(C)** The flight muscles of female crickets on the 1st day after eclosion; **(D)** The anatomy of female crickets on the 10th day after eclosion; **(E)** The abdominal anatomy of female crickets on the 10th day after eclosion; mature eggs were clearly observable after abdominal dissection; **(F)** The flight muscles of female crickets on the 10th day after eclosion.

The pattern of flight muscle histolysis was similar between males and females of *A. domesticus*. The flight muscle histolysis mainly occured on the 9th-11th days after eclosion. In addition, by measuring the length, width and height of the dorsal longitudinal flight muscles of *A. domesticus* individuals from 0 to 11 days after eclosion, we found that the length remained almost unchanged over the degradation process, whereas the width gradually decreased (Supplementary Table S4). To visualized the volume variation from before to after flight muscle histolysis, we divided the degradation process of flight muscle of *A. domesticus* into four stages (Figures 1A, B). The flight muscle had degraded to the greatest extent at Stage IV but had not fully disappeared (Figure 1C). LSD and Tukey’s B^a^ tests were both employed to assess the statistical significance of the DLM volume differences among the four stages of flight muscle histolysis. The results showed no significant difference in DLM volume between Stage I and Stage II, but as expected, there was a significant difference between Stage I and Stage III or Stage IV, with that between Stage I and Stage IV being more obvious (*p* < .05). These results indicate that volume changes significantly during the degradation of the flight muscle and tends to decrease. In addition, the color of the dorsal longitudinal flight muscle changed from pinkish-brown to pale, the morphology changed from full and smooth to flabby and thin, and the volume significantly decreased with flight muscle histolysis (Figure 1; Supplementary Table S4). The abdomen of *A. domesticus* was enlarged after flight muscle histolysis (Figure 2), and eggs were visible, suggesting that flight muscle histolysis is closely related to reproduction in *A. domesticus*.

### 3.2 Transcriptome analyses of *A. domesticus* before and after flight muscle histolysis

#### 3.2.1 Sequence analysis and assembly of flight muscle transcriptome

The flight muscle transcriptome of *A. domesticus* was sequenced, and 13.58 Gb of high-quality, clean read data were obtained after quality control; the percentage of Q30 bases was 94.53%. To completely assemble the low-abundance transcripts, samples from the two stages (O1 and R1) were combined. Transcriptome assembly was accomplished based on the left.fq and right.fq files obtained using Trinity (Grabherr et al., 2011) with min_kmer_cov set to 2 by default and all other parameters set to default. In total, 66,241 transcripts and 36,163 unigenes were assembled. The N50 length of the transcripts was 3,350 bp, the N50 length of the unigenes was 2,274 bp, and the assembly was largely complete. A total of 36,163 unigenes were filtered; of these, 12,096 unigenes had a length greater than or equal to 1 kb (Supplementary Table S5; Supplementary Figure S1). The clean data from the two stages were compared with the assembled unigene library (Supplementary Table S6).

#### 3.2.2 Functional annotation of genes in the flight muscle transcriptome

A total of 13,449 unigenes were annotated in the flight muscle transcriptome of *A. domesticus*. Among them, 13,246 unigenes were annotated by the NR database, which accounted for the highest proportion of unigene annotations. The number of unigenes annotated by each database is shown in Supplementary Table S7. Comparison of the FPKM values of the samples between the two stages revealed that the FPKM median line of stage R1 (day 10 after eclosion) was higher than that of stage O1 (day 0 after eclosion) (Supplementary Figure S2).

#### 3.2.3 Analysis of DEGs before and after flight muscle histolysis in *A. domesticus*

**FIGURE 3:**
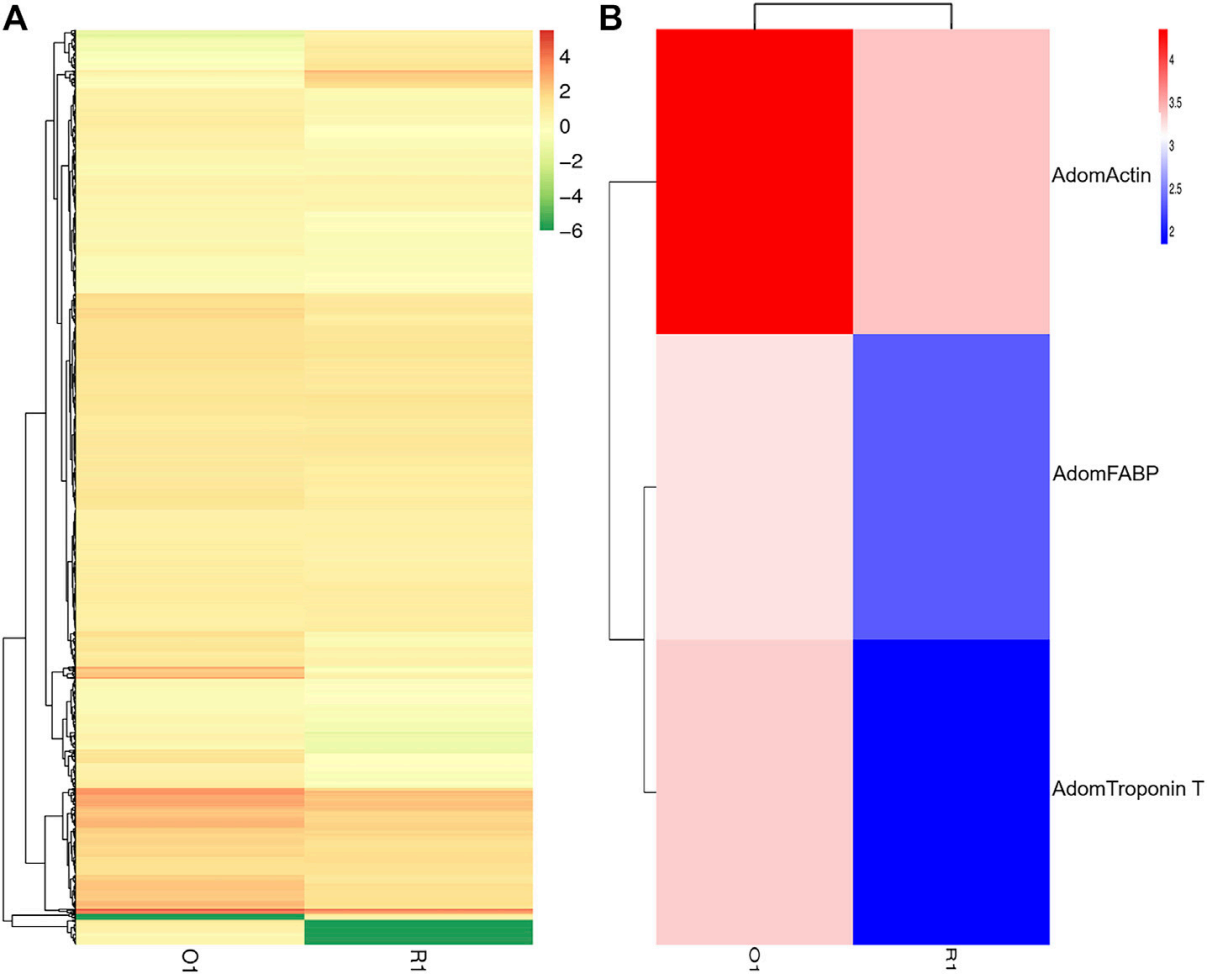
Analysis of DEG between before and after flight muscle histolysis in *A. domesticus*. **(A)** Heatmap of the DEGs between before and after flight muscle histolysis in *A. domesticus*. **(B)** Heatmap of *AdomFABP*, *AdomTroponin T* and *AdomActin* expression based on their FPKM values in the two stages in *A. domesticus*.

To investigate the expression of DEGs between before and after flight muscle histolysis in the flight muscle samples from *A. domesticus*, the samples from each stage were compared with those from the other. Prior to differential gene expression analysis, for each sequenced library, the read counts were adjusted by the EBSeq package through an empirical Bayesian approach. Differential expression analysis of two samples was performed using the EBSeq R package. *p*-value < .01 and |log2 (fold change) |>2 was set as the thresholds for significantly differential expression. The results of volcano plots (Supplementary Figure S3A), MA plots (Supplementary Figure S3B) and the heatmap plot (Figure 3) showed that most DEGs were downregulated after flight muscle histolysis. The genes that had altered expression are likely to be key genes in the regulation of flight muscle histolysis in *A. domesticus*. Thus, in this experiment, key genes involved in the regulation of flight muscle histolysis in *A. domesticus* were screened by focusing on DEGs whose expression changed significantly after flight muscle histolysis.

**FIGURE 4:**
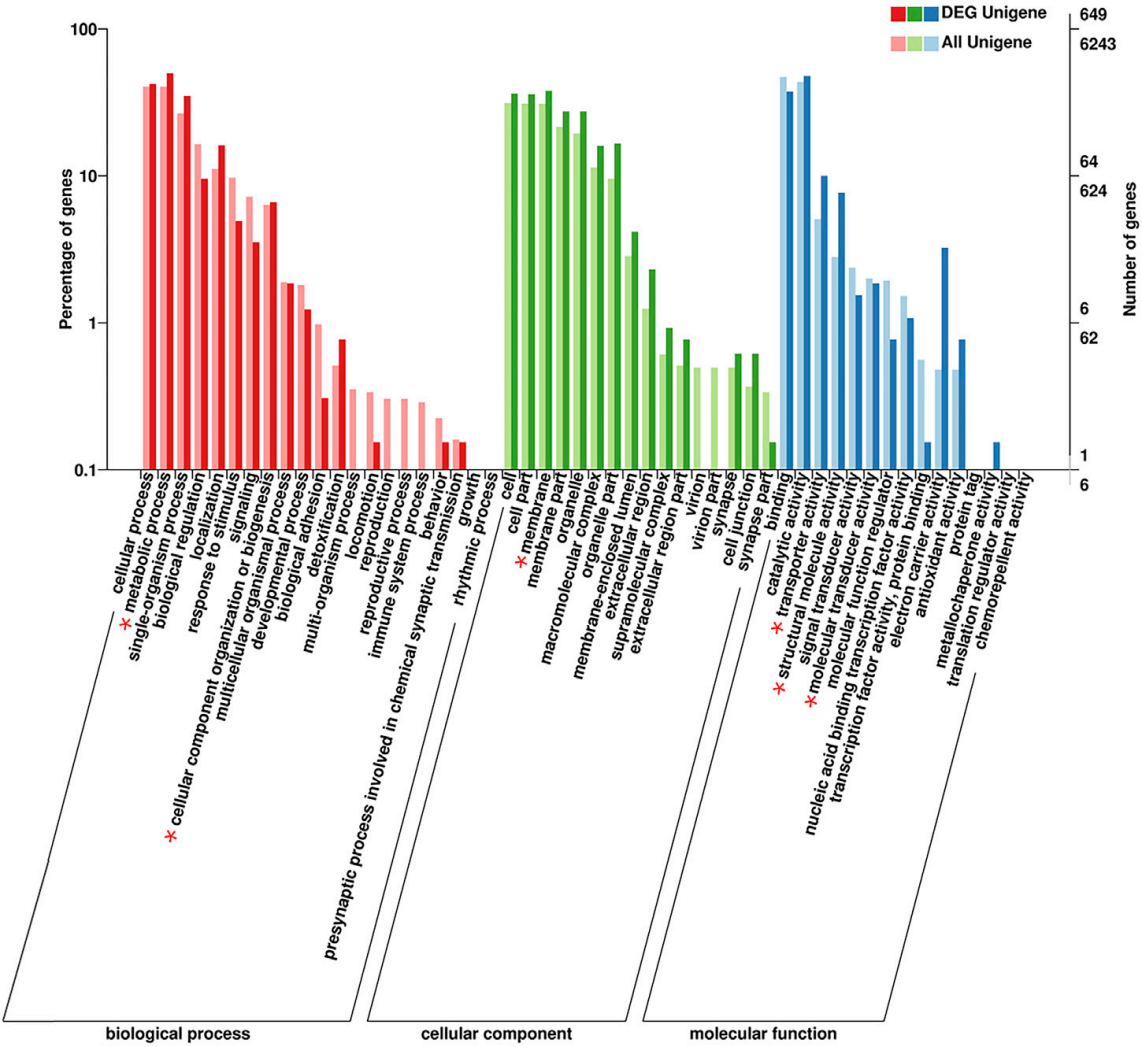
GO classification of DEGs related to flight muscle histolysis in *A. domesticus*.

In total, 1,685 DEGs were identified between the two samples, including 118 upregulated genes and 1,567 downregulated genes. To clarify the types and functions of these significantly altered genes, GO analysis was applied to the DEGs related to flight muscle histolysis for functional annotation; other analyses included COG direct homology classification, eggNOG direct homology classification, and KEGG functional annotation. GO functional annotation showed that the DEGs were mainly related to the growth and development of flight muscles (Figure 4). Both results of the COG (Supplementary Figure S4A) and eggNOG (Supplementary Figure S4B) direct homology classification analyses indicated that insect flight energy production, transformation and metabolic activities were increased after flight muscle histolysis in *A. domesticus*. The KEGG functional analysis showed that a large number of DEGs were involved in ribosomal activity during flight muscle histolysis and in the metabolism of sugars, lipids and proteins. In addition, a large number of DEGs were involved in the “fatty acid metabolism” (5.05%) and “fatty acid degradation” (4.73%) pathways, indicating that the fatty acid pathway may play an important role in flight muscle histolysis (Supplementary Figure S5A). The KEGG scatter plot showed that DEGs were mainly enriched in the “fatty acid metabolism”, “biosynthesis of amino acids”, “ribosome”, “fatty acid degradation” pathways and so on (Supplementary Figure S5B). In summary, the GO, COG, eggNOG and KEGG functional analyses of DEGs revealed that the DEGs were mainly involved in pathways related to growth and development, the production and transformation of energy-related substances, protein synthesis and metabolic interactions. The findings show that the analysis of DEGs is a reasonable and feasible entry point for screening candidate genes and identifying pathways related to flight muscle histolysis in *A. domesticus*.

### 3.3 Screening and functional verification of genes regulating flight muscle histolysis in *A. domesticus*

#### 3.3.1 Screening of candidate genes involved in flight muscle histolysis

The top 200 upregulated genes and top 200 downregulated genes were focused on to identify the DEGs involved in flight muscle histolysis of *A. domesticus*. Based on the genes related to flight muscle development mentioned in existing studies and combined with the analysis results of differences in expression levels, finally decided to screen these genes first from three aspects: fatty acid, troponin and Actin. According to the results of the National Center for Biotechnology Information (NCBI) BLAST and genes functional annotation, three candidate genes were identified, namely, *AdomFABP*, *AdomTroponin T* and *AdomActin* (Supplementary Table S8).

#### 3.3.2 Expression levels of the candidate genes before and after flight muscle histolysis

**FIGURE 5:**
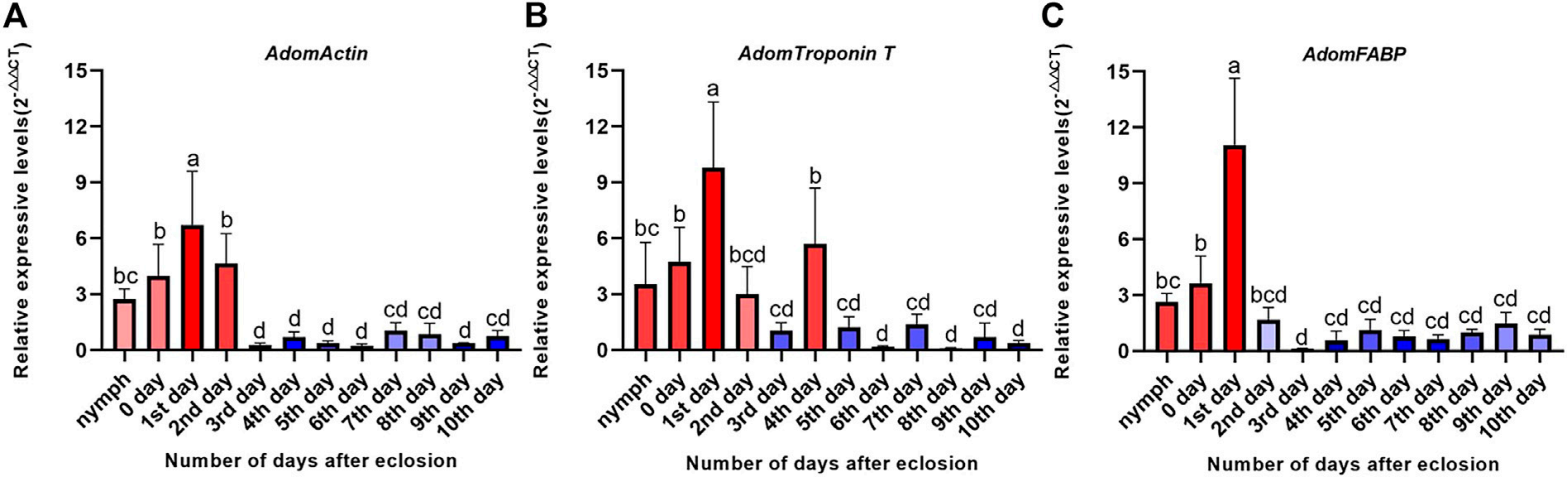
Expression levels of three candidate genes during flight muscle development in *A. domesticus*. **(A)** Expression level of *AdomActin* in 12 periods; **(B)** Expression level of *AdomTroponin T* in 12 periods; **(C)** Expression level of *AdomFABP* in 12 periods. Note: The data were analyzed by LSD and Waller-Duncana, b (*p* < .05), and the letters a/b/c/d were used to indicate significance. “nymph” denotes the last nymph age of *A. domesticus*, and the 0–10th days denote the 0–10th days after eclosion.

The RT-qPCR results of the three candidate genes showed that the expression levels of the candidate genes were significantly different between before and after flight muscle histolysis, with those from before histolysis being significantly higher (Figure 5). The expression trends of the candidate genes on days 0 and 10th were consistent with the results of the transcriptomic analysis. In addition, RT-qPCR results showed that the expression of the three candidate genes was upregulated in nymphs within 0–1 days after molting to adulthood. *AdomFABP* was most downregulated on the 2nd day (Figure 5C), which was higher than the other two candidate genes *AdomActin* and *AdomTroponinT* (Figures 5A, B), indicating that AdomFABP might play a key role in the degradation signal transduction pathway.

#### 3.3.3 Detection of RNAi results by RT-qPCR

**FIGURE 6:**
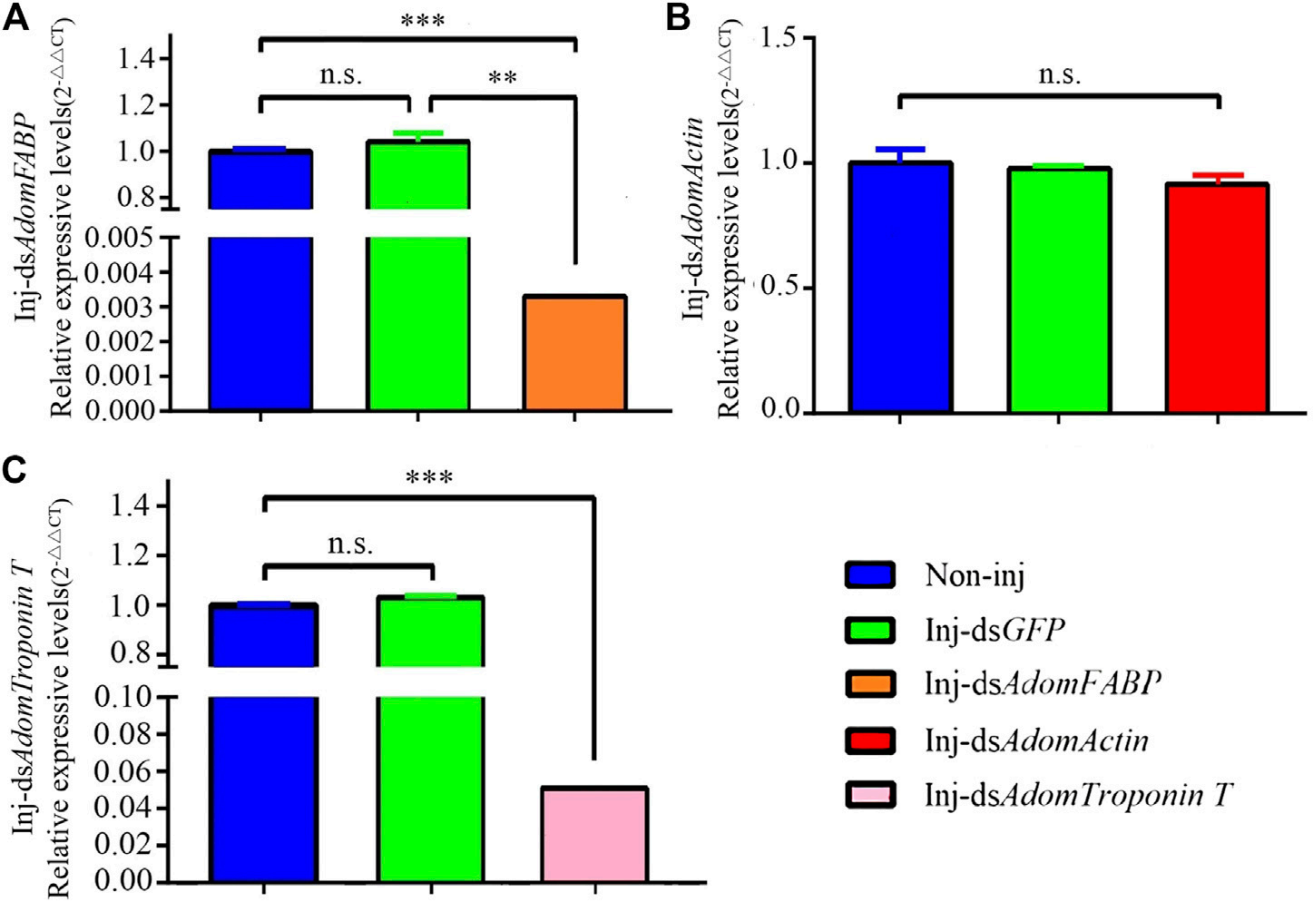
RT-qPCR results after RNAi in *A. domesticus*. **(A)** The expression of *AdomFABP* after RNAi; **(B)** The expression of *AdomActin* after RNAi; **(C)** The expression of *AdomTroponin T* after RNAi. Note: T tests were used to analyze gene expression differences; n.s. indicates no significant difference, ** indicates *p* < .01, and *** indicates *p* < .001. The figure displays the mean value, N = 3.

To further assess whether the three candidate genes play regulatory roles in flight muscle histolysis in *A. domesticus*, the expression of *AdomFABP*, *AdomTroponin T* and *AdomActin* was knocked down by RNAi. On the 6th day of dsRNA injection, candidate gene expression was detected (Figure 6). Compared with the GFP and wild-type groups, the gene injected with ds*AdomFABP* and ds*AdomTroponin T* exhibited significantly lower expression of the corresponding genes (*p* < .01) (Figures 6A, C); the expression of the third candidate gene (*AdomActin*) was not significantly altered in the treated group (*p* > .05), nor was its expression significantly changed in the negative control dsGFP treatment group (*p* > .05) (Figure 6B). Thus, the interference of *AdomFABP* and *AdomTroponin T* was successful. Compared with those of the GFP and wild-type groups, the body condition, growth and development, and physiological status of *A. domesticus* remained unchanged after dsRNA injection (Supplementary Figure S6). This result indicated that the injection volume and quantity in the RNAi experiment were sufficient.

#### 3.3.4 Flight muscle histolysis of *A. domesticus* after RNAi

**FIGURE 7:**
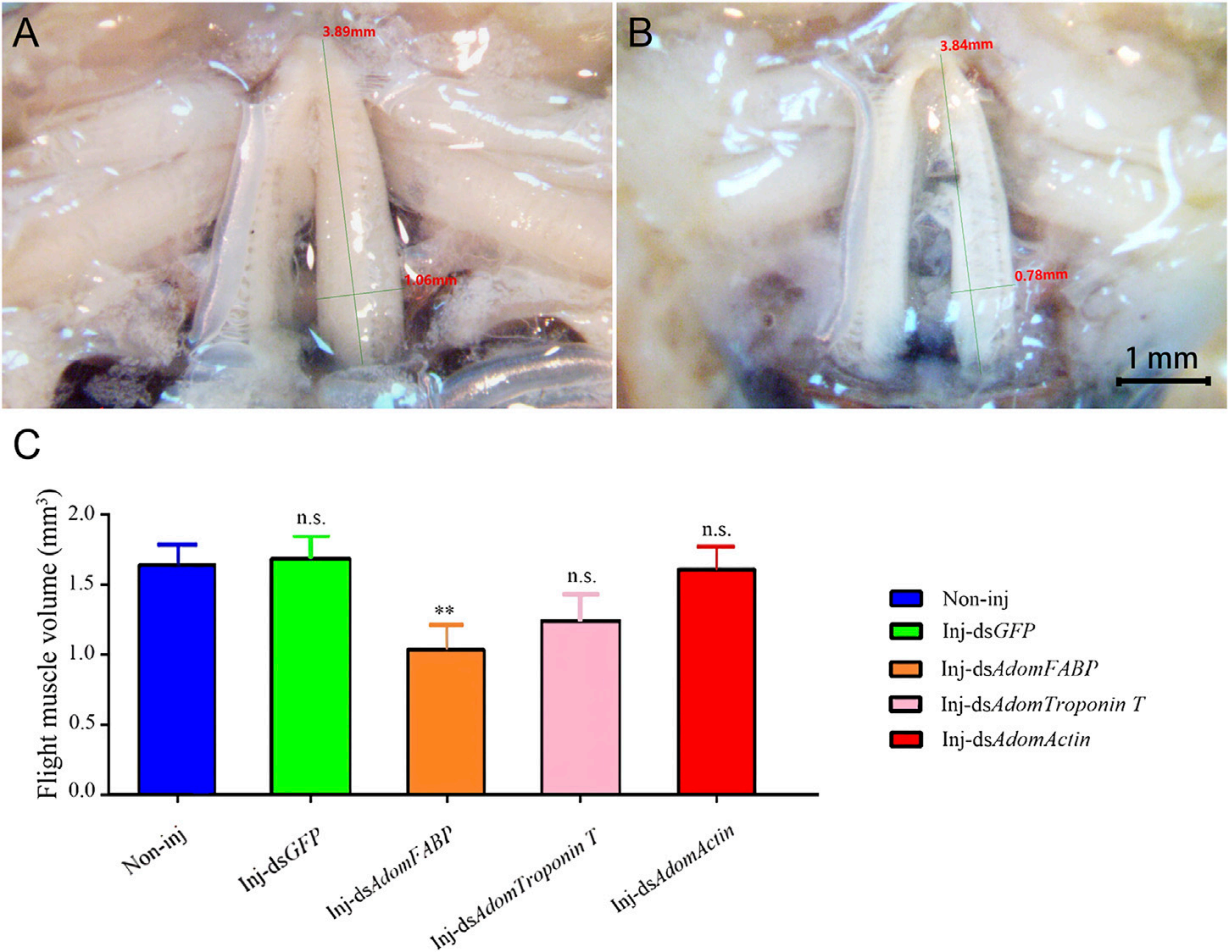
Comparison of flight muscle histolysis of *A. domesticus* on the 6th after RNAi. **(A)** The flight muscle morphology of wild-type; **(B)** The flight muscle morphology after ds*AdomFABP* treatment; **(C)** Comparison of flight muscle volume after RNAi.

After RNAi for GFP and the three candidate genes in adult crickets, flight muscle histolysis in each group was anatomically observed from day 3 to day 7. On the 6th after dsRNA injection, flight muscle was significantly degraded in the ds*AdomFABP*-injected group compared with the wild-type (Figures 7A, B) and dsGFP-injected groups (*p* = .0031). This result coincides with the DEG analysis showing that *AdomFABP* was downregulated when flight muscle histolysis occurred in house crickets. Thus, the results of both the transcriptome and RNAi assays revealed that *AdomFABP* was involved in the molecular regulation of flight muscle histolysis of *A. domesticus*. However, there was no significant change in flight muscle histolysis in the group injected with ds*AdomTroponin T* (*p* = .1461) or the group injected with ds*AdomActin* (*p* = .8818) (Figure 7C).

## 4 Discussion

Flight muscle histolysis is carried out under the control of genes and is a complex process involving multiple genes and multilevel regulation. Although researchers have cloned and analyzed the structural genes and related regulatory genes of flight muscles in various insects, such as fruit flies (Nongthomba et al., 2001), plant hoppers (Vellichirammal et al., 2014), aphids (Kobayashi and Ishikawa, 1994; Ishikawa et al., 2008), and locusts (Liang et al., 2020), but the molecular mechanism of flight muscle tissue degradation has not been fully elucidated, and many related scientific problems have not yet been addressed. Additionally, beyond research in the fruit fly, which is a model insect employed for the study of flight muscle histolysis because its thoroughly studied genome facilitates the application of gene silencing and other technologies, the study of flight muscle histolysis has mainly focused on insects with migratory ability. However, there are many species of insects that do not have the ability to migrate, but in which flight muscle will degrade to meet the needs of growth and development or adaptation to the environment. The house crickets are one of them, in which the flight muscle histolysis after eclosion is widespread in the population.

Compared with longitudinal flight muscles, transverse flight muscles show no obvious morphological changes during flight muscle histolysis; thus, we focused only on the DLMs in this study. Flight muscle histolysis was observed in *A. domesticus* by dissecting out the DLM and then measuring its length, width and height. The observed changes in the color and morphology of flight muscle were consistent with those of Oliver et al. (2007). In addition, after measuring the wet weight of the tissue, total protein and percentage muscle shortening in the DLM of *A. domesticus*, Oliver et al. (2007) noted that the flight muscle histolysis in *A. domesticus* was complete on 3rd day after adults eclosion. Notably, in the present study, the peak flight muscle histolysis occurred on the 9th to 11th days after eclosion. This difference between studies may be related to study differences in breeding conditions. The feeding temperature and photoperiod in Oliver et al. (2007) were respectively 30°C and L:D = 14:10, but those of this study were 30°C and L:D = 0:24. This could be because environmental factors influence the timing of flight muscle histolysis (Roff, 1990), which in turn affects the pattern of flight muscle histolysis. There was no sexual dimorphism in the peak time of flight muscle histolysis in the crickets. However, the abdomens of females began to swell after flight muscle histolysis, and eggs were visible after dissection. These findings might provide insights into the trade-off between flight muscle maintenance and reproductive activity (Tanaka, 1993).

In this study, through different enrichment analysis of DEGs, it was found that most differentially expressed genes are enriched in the pathway related to energy metabolism, which is consistent with the strong energy metabolism function of flight muscle. Of which, COG and eggNOG enrichment analysis results was performed that many DEGs were enriched in the “Lipid pathway transport and metabolism” pathway; KEGG enrichment analysis also showed that relative majority DEGs were enriched in the “Fatty acid metabolism” and “Fatty acid degradation” pathways. Studies have indicated that fatty acid metabolism is an important metabolic mode for long-distance migration of insects (Arrese and Soulages, 2010; Guo et al., 2022), combined with the pattern of flight muscle histolysis after migration (Feng et al., 2019), it can be inferred that compared with other metabolic pathways, fatty acid metabolic pathway is more likely to be a regulatory pathway involved in and regulating flight muscle histolysis, and fatty acid transporters are more likely to act as regulatory signals than other proteins with fixed metabolic functions due to their properties of transporting fatty acids. In addition, it is reported that FABP has also been reported to be crucial for long-distance migration of migratory locusts (Rajapakse et al., 2019). That is the reason why this study finally focused on it for research.

Our study successfully verified the important regulatory role of AdomFABP in flight muscle histolysis of *A. domesticus*. Downregulation of *AdomFABP* gene expression promotes this process in *A. domesticus*. FABP has been reported to play an important role in lipid uptake and transport in locust flight muscle and is an essential element of skeletal muscle metabolism involved in sustained muscle activity, the absence of which prevents locusts from migrating long distances (Rajapakse et al., 2019). Increased FABP levels are associated with the flight ability of insects (Haunerland, 1997). Flight activity can promote FABP expression in adult locusts, and the expression of FABP in flight muscles has been shown to be upregulated before adults commenced long-distance migration (Chen and Haunerland, 1994). The flight ability of insects mainly depends on the development of their flight muscle structures (Ding et al., 2014). FABP plays an important role in the energy supply of flight muscles and affects flight muscle formation and degradation (Wu et al., 2002). Therefore, it can be inferred that the degradation of flight muscle reduces or prevent flight ability and that the expression of FABP in flight muscle inevitably decrease as well, which supports the results of this study. In addition, in the present study, the expression level of *AdomFABP* was compared between before and after flight muscle histolysis based on RT-qPCR. The results showed that the expression of *AdomFABP* was upregulated in nymphs within 0–1 days after molting to adulthood, indicating that the flight muscles continued to develop and remained intact within 0–1 days after adulthood. However, when crickets entered the 2nd day of adulthood, the expression of *AdomFABP* was significantly downregulated, suggesting that *AdomFABP* might play a role in the degradation of flight muscle by inhibiting muscle development. Similar to the house cricket, many species of locusts show wing dimorphism (with long-winged and short-winged morphs). Wing dimorphism has been proposed as a strategy to balance trade-offs in ecological systems between flight capability and various fitness components (Chen et al., 2019). Long-winged individuals show better migration ability than short-winged individuals, and the transition from the long-winged to the short-winged form is accompanied by flight muscle histolysis. Therefore, the study of flight muscle histolysis in *A. domesticus* could lay a theoretical foundation for the study of wing dimorphism and migration behavior in locusts. However, FABP is a binding protein; thus, the factors that decrease its expression and thereby regulate flight muscle histolysis in *A. domesticus,* as well as other genes and proteins in the FABP pathway that jointly regulate flight muscle histolysis in this insect, need to be identified in future studies.

AdomTroponin and AdomActin may also be involved in flight muscle histolysis. Troponin consists of three subunits, troponin C (TpnC), troponin I (TpnI) and troponin T (TpnT). The different physiological functions of insect muscles can be attributed, at least in part, to specific troponin subunits, and it has been suggested that the acquisition of flight muscle functions (e.g., contraction) may be associated with these subunits (Liang et al., 2020). Actin is a multifunctional protein that regulates muscle contraction. Degradation of actin into two molecular fragments of different sizes can lead to disruption of cell viability and eventually to apoptosis (Mashima et al., 1997); this mechanism is generally considered to be the main mode of flight muscle histolysis. Thus, it is clear that *Actin* is involved in the regulation of flight muscle histolysis. Additionally, *ActinAct88F* is specifically expressed in the indirect flight muscle of adult *Drosophila melanogaster*, and mutations in this gene result in reduced flight ability (Mogami and Hotta, 1981). However, in the present study, when RNAi was used to verify the function of *Troponin T*, it was found that although *Troponin T* was successfully knocked down, the morphology of *A. domesticus* flight muscle was not affected (Figures 6, 7). This result suggests that AdomTroponin T plays only an indirect regulatory role in flight muscle histolysis in *A. domesticus* or has nothing to do with flight muscle histolysis. The RNAi knockdown of AdomActin was not successfully realized, may be due to the special status of AdomActin in flight muscle, which was not easy to be interfered successfully. In addition, it is also possible that the location selection of dsRNA fragments is not appropriate. Therefore, whether the *Actin* is involved in the regulation of flight muscle histolysis further interference and functional studies of *Actin* are needed in the future.

At the hormone level, it has been found that juvenile hormones, prothymosin, etc., are involved in flight muscle histolysis (Feng et al., 2019), but the genes and related pathways involved in the regulation of flight muscle histolysis remain unknown. We will focus on further analyzing the intrinsic regulatory mechanism of flight muscle histolysis in the future. This study can serve as a reference for the future explorations of the molecular mechanism and physiological significance of flight muscle histolysis in Orthoptera.

## Data Availability

The data presented in the study are deposited in the Sequence Read Archive repository, accession number: SRR22200957 and SRR22200956. The data link: https://www.ncbi.nlm.nih.gov/sra/?term=SRR22200956; https://www.ncbi.nlm.nih.gov/sra/?term=SRR22200957.
