# Supplementary material for: Screening and identification of genes associated with flight muscle histolysis of the house cricket Acheta domesticus
Source: Front Physiol. 2023 Jan 11;13:1079328. doi: 10.3389/fphys.2022.1079328 (PMC9873970; doi:10.3389/fphys.2022.1079328)
Supplement: Supplementary file 2 [file Table1.docx]

Supplementary Material

# Supplementary Table 1. Primers of candidate genes used for RT-qPCR assay.

| **Gene name** | **Sense primer** | **Antisense primer** |
| --- | --- | --- |
| *AdomFABP* | TACATGAAGGCGATGGGCGT | CCGTGTTCTTGAAAGTGGAGGT |
| *AdomTroponin T* | AGGGTCCAAACTTCACCATCA | GGTACGCAGCTTATCAACACTCA |
| *AdomActin* | GCCATCACCAGAGTCCAACA | AGGCCAACCGTGAGAAGATG |
| *18s rRNA* | CAGTCGTGACCCGAAAGG | ATCAGCCCAAGGTTATCCAG |
