# Supplementary material for: Screening and identification of genes associated with flight muscle histolysis of the house cricket Acheta domesticus
Source: Front Physiol. 2023 Jan 11;13:1079328. doi: 10.3389/fphys.2022.1079328 (PMC9873970; doi:10.3389/fphys.2022.1079328)
Supplement: Supplementary file 3 [file Table8.docx]

Supplementary Material

**Supplementary Table 8.** Related sequence screening.

| **No.** | **Unigene ID** | **Annotation** | **Amino acid sequence** |
| --- | --- | --- | --- |
| 1 | c21367.graph_c0 | fatty acid-binding protein (*Camponotus floridanus*) | MEELYGKQYKLNKSEKFDEYMKAMGVGMMTRKLGGTVSPVIELTEADGVYTLKSTSTFKNTVISFKLGEEFDEETPDGRKVKSTITRDGNKLIQIQKGEPKDTEIIREFSPEQVLMTLKVDDIVCTRIYKAI |
| 2 | c10980.graph_c0 | troponin T (*Teleogryllus emma*) | MSDDEDVYSEEEEEVEVKPRKSIVGDQPQGDPEFIKRQEQKSSALDEQLKEYIAEWRKQRSKEEDELKRLKEKQAKRKVMRADEEKRLAERKKQEEERRQREIEEKKQRDIEEKRRRLEEAERKRQAMMQAMKEQNKKGPNFTITKKDQSFNLSSAQIERNKTKEQLEEEKKISLSFRIKPLEIEGLSVDKLRTKATELWEAIVKLETEKYDLEERQKRQDYDLKELKERQKQQLRHKALKKGLDPEALTGKYPPKIQVASKYERRVDTRSYDDKKKLFEGGWATLSSEVNEKVWKSKYEVFTNRTKSKLPKWFGERPGKKKGDPESPEEEEVKAPGADDDLDEPTFEPEPEPEPEEEEVEEEEEEEEEEEEEEEEEEEE |
| 3 | c16538.graph_c1 | actin (*Acyrthosiphon pisum*) | MVSASSLFPWVVVSAVAAAGGCCCLEALAVHNARAGLIVLLLGDPHLLEGGQGSQDGAADPDGVLPLRGSDDLDLDGAGSQGGDFLLHPVSNTGVHGGATGQDSVGVQVLTDVDVALHDGVVDGLVDAAGFHTQEGRLEEGLRAAETLVADGDDLAVGQLVGLLQGGGGGGGGHFLLEVQGDVAQLFLDVAHDFTLGSGGEAVATLSQDLHQVVGEIAASQVQTQDSVGQSITFIDGDGVGDAITRVQHNTSGTAGSVQGQHGLDGDVHGGGVEGLKHDLGHLLTVGLGVQGSLSEQDGVLLGGHTQLIVEGVMPDLLHVVPVGDDAVLNGVLQGEDTSLGLGLITNVAVLLTHTHHHTLMAGPADDGREDGAGSVVSGEAGLAHAGAIVHDQSSNVVVTHFGRWLLFRASLLLAPALLLDV |
