# Supplementary material for: Screening and identification of genes associated with flight muscle histolysis of the house cricket Acheta domesticus
Source: Front Physiol. 2023 Jan 11;13:1079328. doi: 10.3389/fphys.2022.1079328 (PMC9873970; doi:10.3389/fphys.2022.1079328)
Supplement: Supplementary file 4 [file Image6.pdf]

# *Supplementary Material*

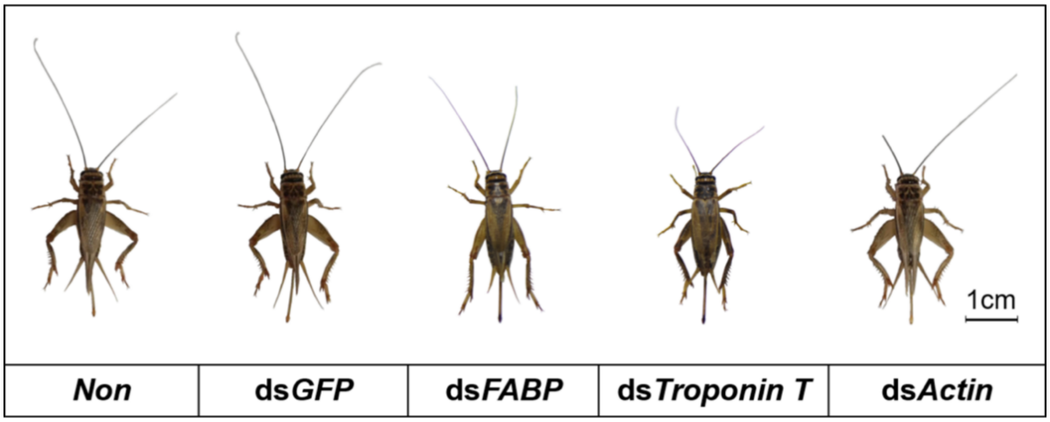


**Supplementary Figure 6.** Female phenotypes after injection of Non, ds*GFP*, ds*FABP*, ds*Troponin T*, and ds*Actin*.
