# Supplementary figures and images for: Screening and identification of genes associated with flight muscle histolysis of the house cricket Acheta domesticus
Source: Front Physiol. 2023 Jan 11;13:1079328. doi: 10.3389/fphys.2022.1079328 (PMC9873970; doi:10.3389/fphys.2022.1079328)

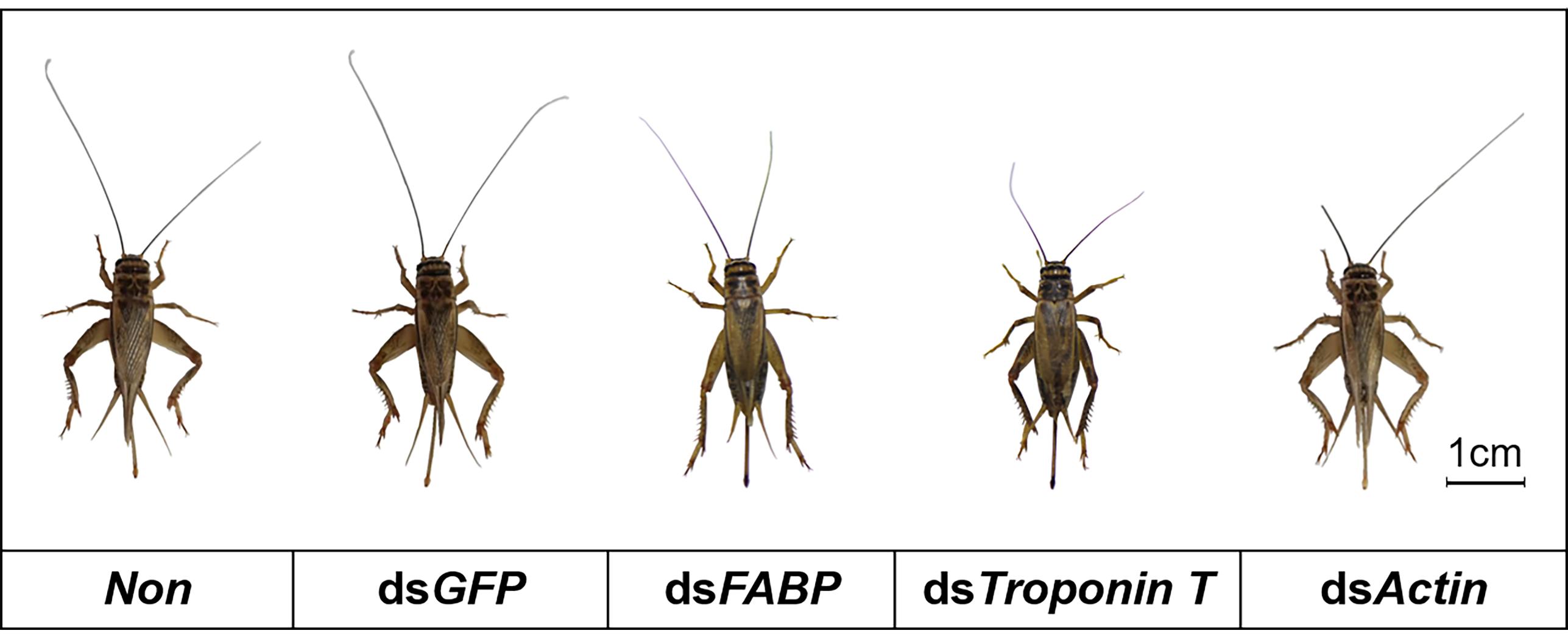

Supplement: Supplementary file 5 [file DataSheet1.ZIP › APPENDIX FIGURE/APPENDIX FIGURE 6.tif]

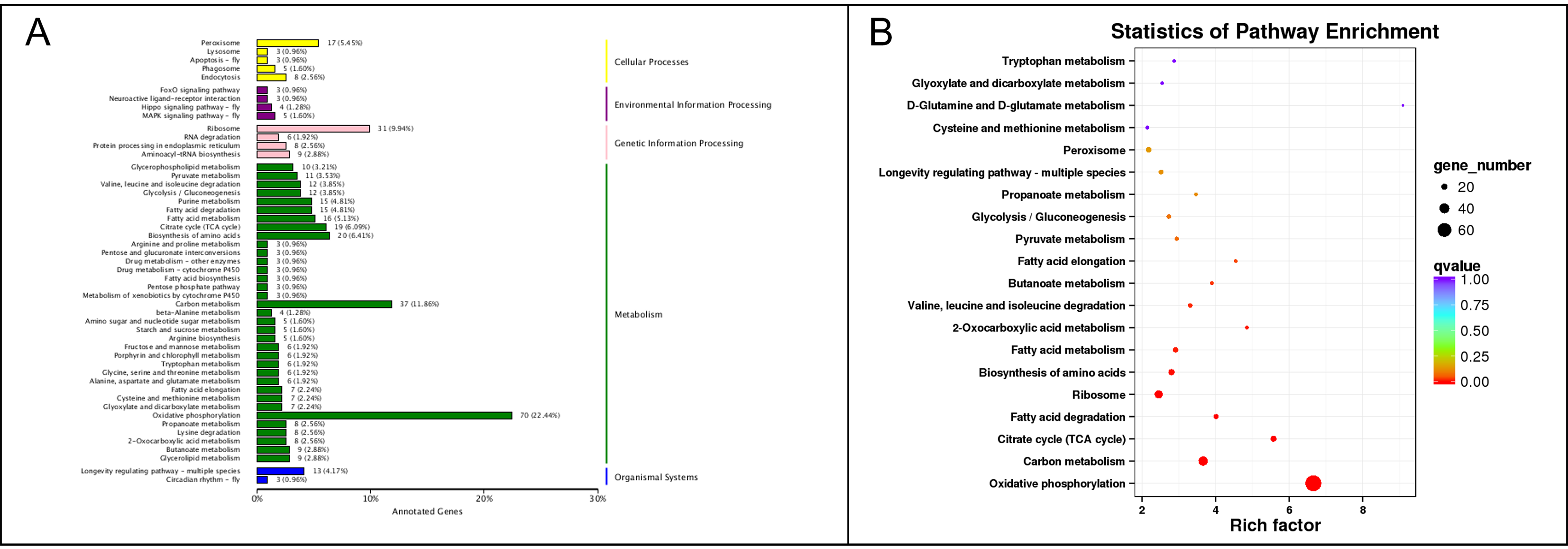

Supplement: Supplementary file 5 [file DataSheet1.ZIP › APPENDIX FIGURE/APPENDIX FIGURE 5.tif]

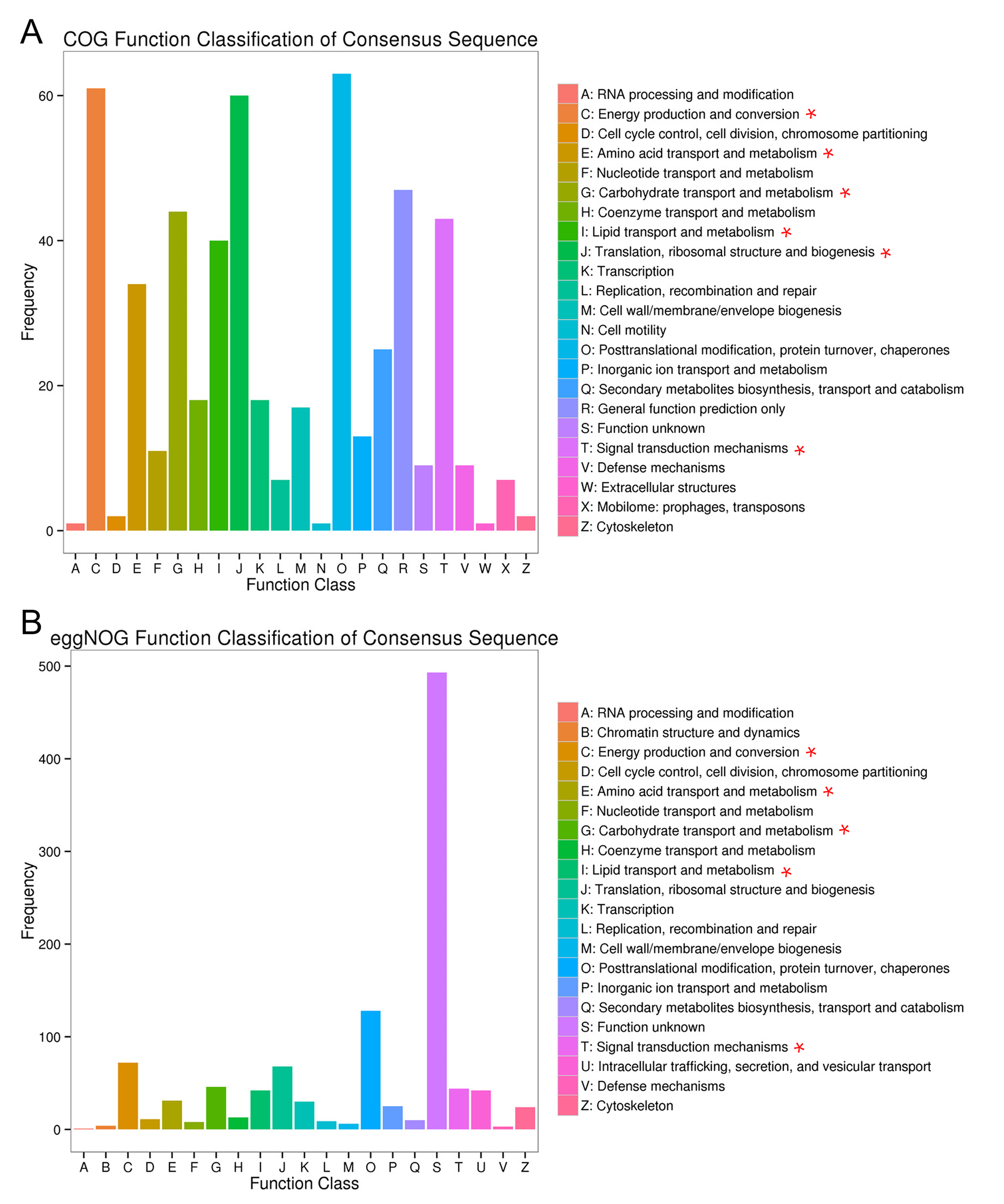

Supplement: Supplementary file 5 [file DataSheet1.ZIP › APPENDIX FIGURE/APPENDIX FIGURE 4.tif]

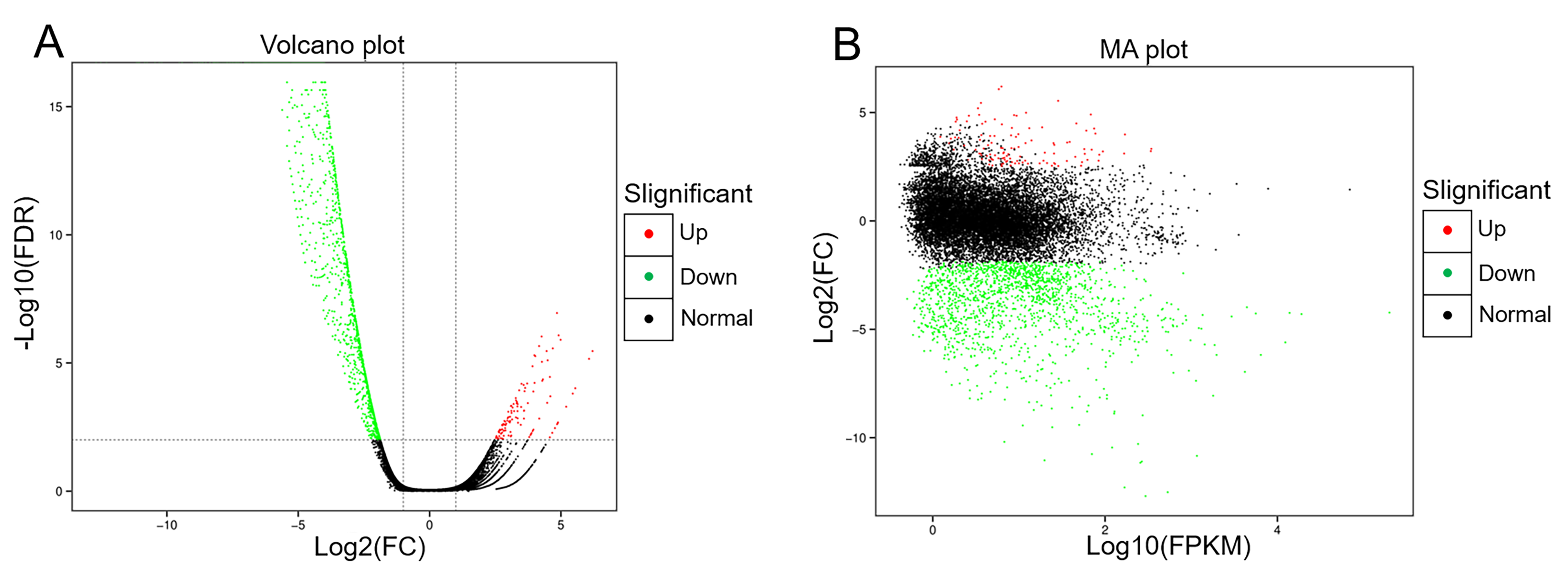

Supplement: Supplementary file 5 [file DataSheet1.ZIP › APPENDIX FIGURE/APPENDIX FIGURE 3.tif]

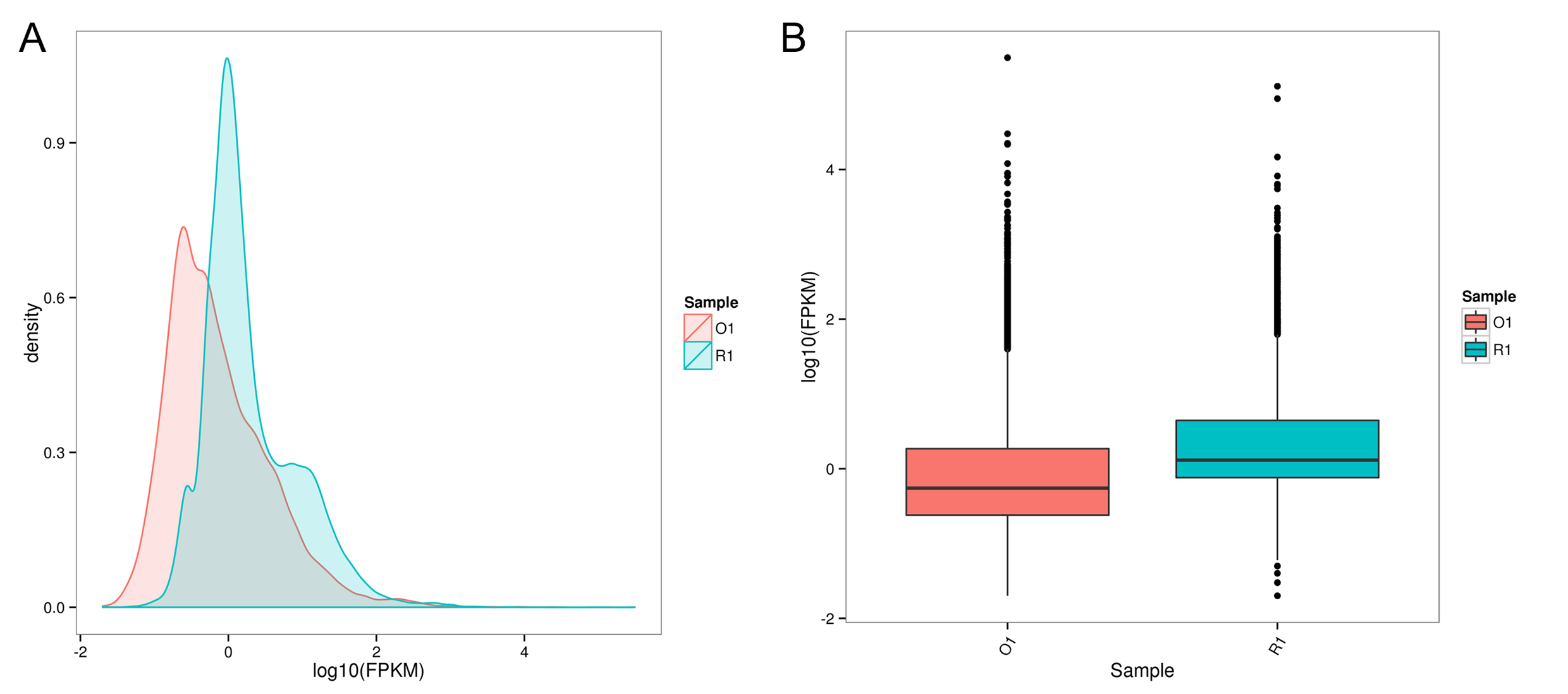

Supplement: Supplementary file 5 [file DataSheet1.ZIP › APPENDIX FIGURE/APPENDIX FIGURE 2.tif]

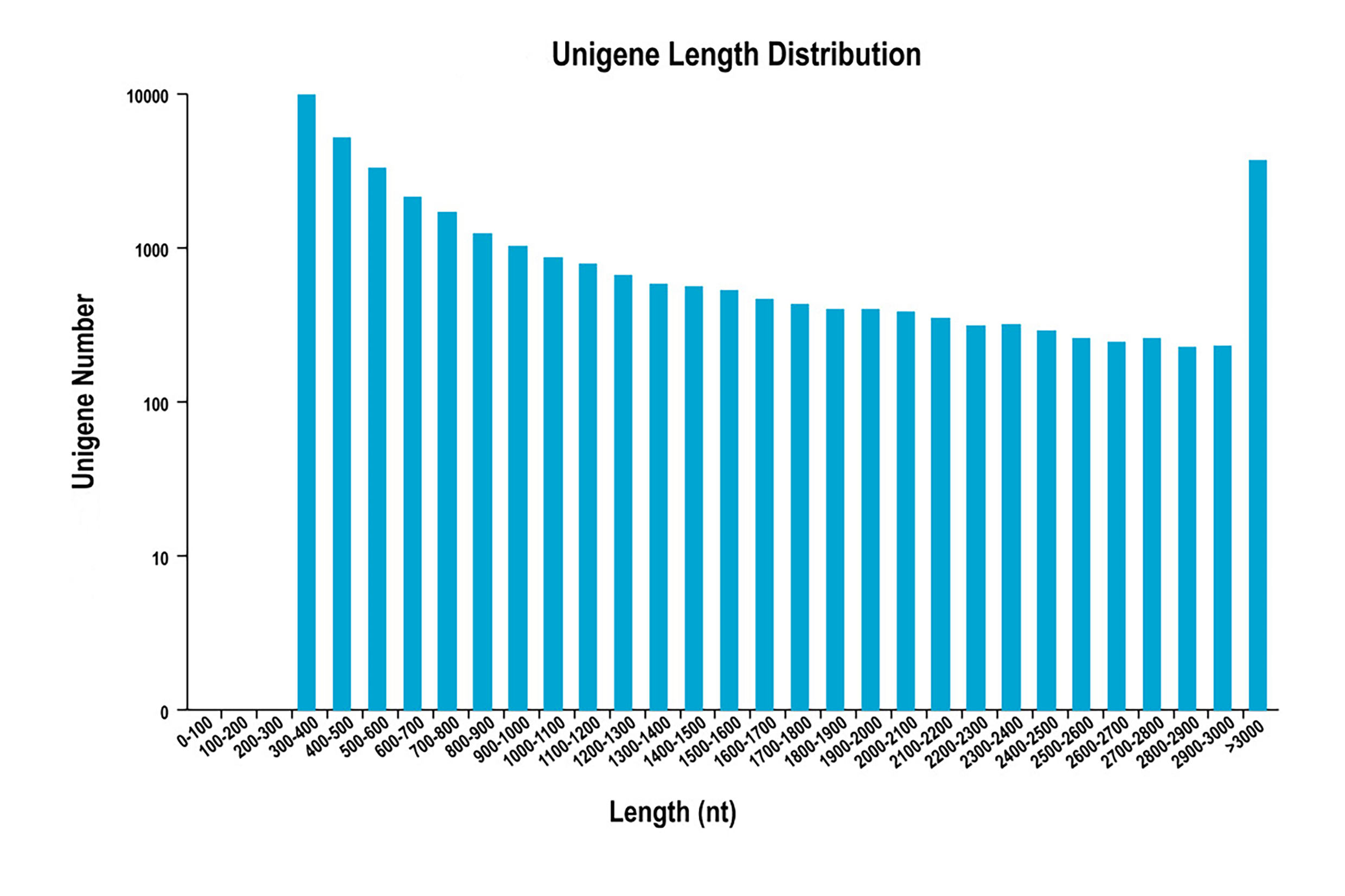

Supplement: Supplementary file 5 [file DataSheet1.ZIP › APPENDIX FIGURE/APPENDIX FIGURE 1.tiff]

## *Supplementary Material*

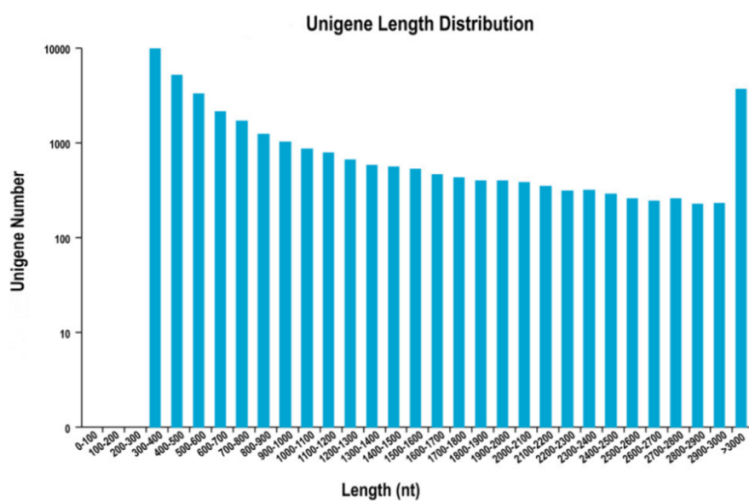

**Supplementary Figure 1.** The distribution of unigene length.

Supplement: Supplementary file 15 [file Image1.pdf]
