# Supplementary material for: Screening and identification of genes associated with flight muscle histolysis of the house cricket Acheta domesticus
Source: Front Physiol. 2023 Jan 11;13:1079328. doi: 10.3389/fphys.2022.1079328 (PMC9873970; doi:10.3389/fphys.2022.1079328)
Supplement: Supplementary file 6 [file Table7.docx]

Supplementary Material

**Supplementary Table 7.** Unigene annotation statistics result.

| **Annotation database** | **Annotated number** | **≥ 300 nt** | **≥ 1,000 nt** |
| --- | --- | --- | --- |
| COG | 3,744 | 820 | 2,924 |
| GO | 6,243 | 2,055 | 4,188 |
| KEGG | 6,608 | 1,884 | 4,724 |
| KOG | 8,646 | 2,469 | 6,177 |
| Swiss-Prot | 7,606 | 2,138 | 5,468 |
| eggNOG | 11,916 | 4,157 | 7,759 |
| NR | 13,246 | 5,088 | 8,158 |
| All | 13,449 | 5,226 | 8,223 |
