# Supplementary material for: Screening and identification of genes associated with flight muscle histolysis of the house cricket Acheta domesticus
Source: Front Physiol. 2023 Jan 11;13:1079328. doi: 10.3389/fphys.2022.1079328 (PMC9873970; doi:10.3389/fphys.2022.1079328)
Supplement: Supplementary file 7 [file Image4.pdf]

Supplementary Material

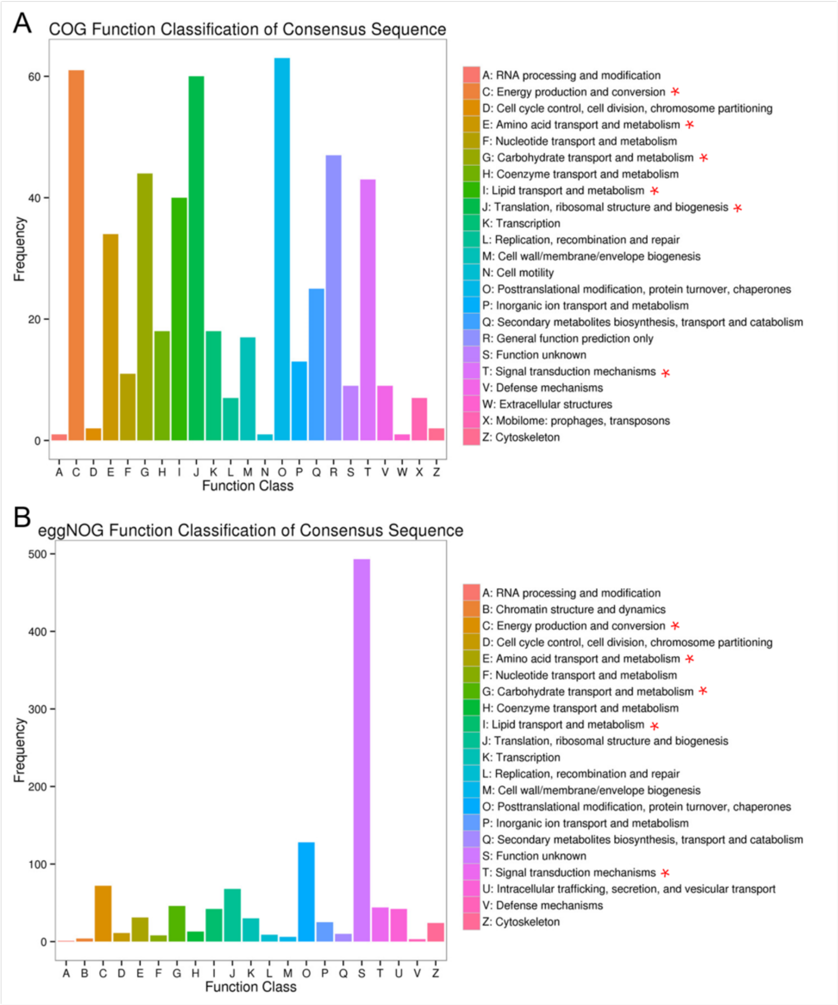

Supplementary Figure 4. DEG direct homology classification according to (A) CGO classification; (B) eggNOG classification.
