# Supplementary material for: Screening and identification of genes associated with flight muscle histolysis of the house cricket Acheta domesticus
Source: Front Physiol. 2023 Jan 11;13:1079328. doi: 10.3389/fphys.2022.1079328 (PMC9873970; doi:10.3389/fphys.2022.1079328)
Supplement: Supplementary file 8 [file Image2.pdf]

Supplementary Material

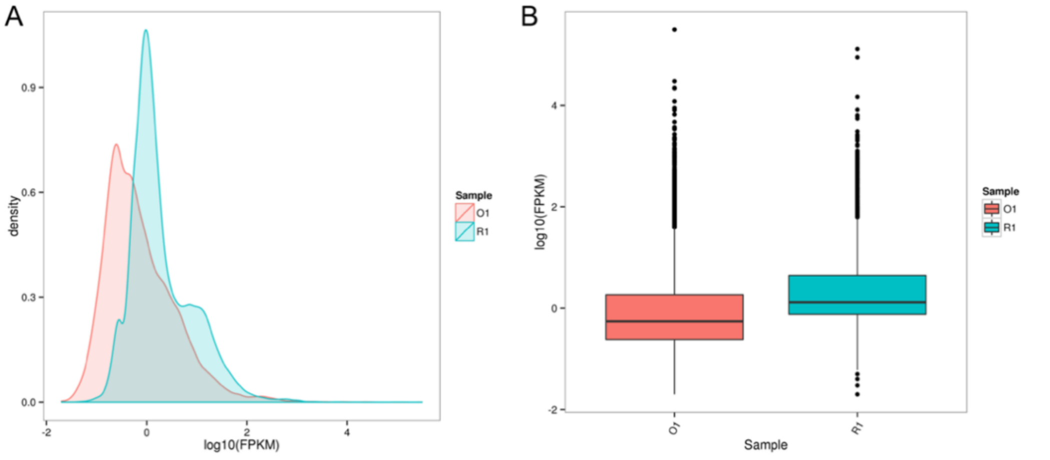

Supplementary Figure 2. General distribution of gene expression before and after flight muscle histolysis in *A. domesticus*. (A) Density distribution curve based on the FPKM values; (B) Box plot of the FPKM values.
