# Supplementary material for: Screening and identification of genes associated with flight muscle histolysis of the house cricket Acheta domesticus
Source: Front Physiol. 2023 Jan 11;13:1079328. doi: 10.3389/fphys.2022.1079328 (PMC9873970; doi:10.3389/fphys.2022.1079328)
Supplement: Supplementary file 9 [file Image3.pdf]

Supplementary Material

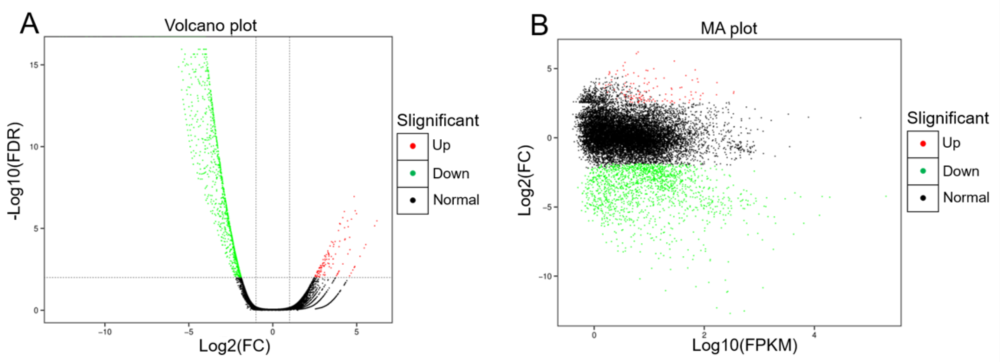

Supplementary Figure 3. The DEG analysis of the comparison between before and after flight muscle histolysis in *A. domesticus*. (A) Volcano plot of the comparison; (B) MA plot of the comparison. Most DEGs were downregulated after flight muscle histolysis.
