# Supplementary material for: Screening and identification of genes associated with flight muscle histolysis of the house cricket Acheta domesticus
Source: Front Physiol. 2023 Jan 11;13:1079328. doi: 10.3389/fphys.2022.1079328 (PMC9873970; doi:10.3389/fphys.2022.1079328)
Supplement: Supplementary file 10 [file Table2.docx]

Supplementary Material

# Supplementary Table 2. Primers of candidate genes used for RNA interference.

| **Gene name** | **Sense primer** | **Antisense primer** |
| --- | --- | --- |
| *AdomFABP* | GTCATCCACCTTCAGAGTC | GAATTGTACGGCAAACAG |
| *AdomTroponin T* | GTAGTTTGGGAAAAGGTTA | AACAACAGTCCAATGAGATA |
| *AdomActin* | CTGGAAGGTGGACAGGGAA | CACTGCCGAGCGTGAAAT |
| *GFP* | GCAGTGCTTCAGCCGCTACCCC | CCCTAGTGAGAGCCGTACCTGCTCG |

# T7: TAA TACGACTCACTA TAGGGAGA was added to the 5’ end of each pair of primers.
