# Supplementary material for: Screening and identification of genes associated with flight muscle histolysis of the house cricket Acheta domesticus
Source: Front Physiol. 2023 Jan 11;13:1079328. doi: 10.3389/fphys.2022.1079328 (PMC9873970; doi:10.3389/fphys.2022.1079328)
Supplement: Supplementary file 11 [file Table5.docx]

Supplementary Material

**Supplementary Table 5.** Sequencing assembly statistics for flight muscle of *A. domesticus*.

| **Length (range)** | **Transcripts** | **Unigenes** |
| --- | --- | --- |
| 300-500 | 18,476 (27.89%) | 14,792 (40.90%) |
| 500-1000 | 14,779 (22.31%) | 9,275 (25.65%) |
| 1000-2000 | 13,040 (19.69%) | 5,620 (15.54%) |
| 2000+ | 19,946 (30.11%) | 6,476 (17.91%) |
| Total number | 66,241 | 36,163 |
| Total length | 120,868,410 | 44,114,064 |
| N50 length | 3,350 | 2,274 |
| Mean length | 1824.68 | 1219.87 |
