# Supplementary material for: Screening and identification of genes associated with flight muscle histolysis of the house cricket Acheta domesticus
Source: Front Physiol. 2023 Jan 11;13:1079328. doi: 10.3389/fphys.2022.1079328 (PMC9873970; doi:10.3389/fphys.2022.1079328)
Supplement: Supplementary file 13 [file Table4.docx]

Supplementary Material

**Supplementary Table 4.** The measurement results of length, width and height of the dorsal longitudinal muscle.

|  | **After Eclosion** | | | | | | | | | | | |
| --- | --- | --- | --- | --- | --- | --- | --- | --- | --- | --- | --- | --- |
|  | **0**  **day** | **1^st^**  **day** | **2^nd^**  **day** | **3^rd^ day** | **4^th^ day** | **5^th^ day** | **6^th^ day** | **7^th^ day** | **8^th^ day** | **9^th^ day** | **10^th^ day** | **11^th^ day** |
| Length (mm) | 3.56 | 3.58 | 3.57 | 3.61 | 3.52 | 3.60 | 3.59 | 3.51 | 3.53 | 3.47 | 3.47 | 3.45 |
| Width (mm) | 1.24 | 1.22 | 1.21 | 1.15 | 1.14 | 1.10 | 1.05 | 1.00 | 0.90 | 0.74 | 0.70 | 0.70 |
| Height (mm) | 0.61 | 0.60 | 0.59 | 0.58 | 0.57 | 0.51 | 0.49 | 0.44 | 0.39 | 0.37 | 0.37 | 0.36 |
