# Supplementary material for: Screening and identification of genes associated with flight muscle histolysis of the house cricket Acheta domesticus
Source: Front Physiol. 2023 Jan 11;13:1079328. doi: 10.3389/fphys.2022.1079328 (PMC9873970; doi:10.3389/fphys.2022.1079328)
Supplement: Supplementary file 14 [file Table6.docx]

Supplementary Material

**Supplementary Table** **6.** Comparison of sequencing data and assembly results between the two stages.

| **Stage** | **Clean reads** | **Mapped reads** | **Mapped ratio** |
| --- | --- | --- | --- |
| Before flight muscle histolysis (O1) | 22,891,445 | 21,763,342 | 95.07% |
| After flight muscle histolysis (R1) | 22,561,355 | 20,599,305 | 91.30% |
